# Decreased levels of interleukin 27 in the serum of vitiligo patients^☆^^☆☆^

**DOI:** 10.1016/j.abd.2020.02.005

**Published:** 2020-06-16

**Authors:** Saeed Malek Hosseini, Naser Gholijani, Nooshafarin Chenari, Kurosh Kalantar

**Affiliations:** a Department of Immunology, School of Medicine, Shiraz University of Medical Sciences, Shiraz, Iran; b Autoimmune Diseases Research Center, Shiraz University of Medical Sciences, Shiraz, Iran

**Keywords:** Cytokines, Inflammation, Vitiligo

## Abstract

**Background:**

Vitiligo is a common skin disorder in which melanocytes are destroyed by auto-reactive immune responses. The loss of melanocytes results in the appearance of depigmented areas in different parts of the body. Cytokines have remarkable roles in the pathogenesis of vitiligo, such as IL-1, IL-6, and TNF-α; interleukin 27 (IL-27) is a new member of the IL-6/IL-12 family, mainly released by activated antigen-presenting cells. IL-27 has been suggested to function as a pro-inflammatory as well as an anti-inflammatory cytokine. Altered concentrations of IL-27 have been shown in various auto-immune diseases such as multiple sclerosis, rheumatoid arthritis, and psoriasis. No studies have been conducted to determine the expression of this cytokine in vitiligo patients.

**Objective:**

The objective of this study was to determine the serum concentration of IL-27 in vitiligo patients and compare it with normal individuals.

**Methods:**

The serum concentration of IL-27 in 79 vitiligo patients was evaluated in comparison to 45 healthy controls using ELISA assay.

**Results:**

Results showed decreased concentration of IL-27 in vitiligo patients as compared with healthy subjects (*p* = 0.026). Furthermore, no correlation between IL-27 concentrations and disease parameters such as vitiligo severity and the extension of the depigmented area was observed.

**Study limitation:**

A larger sample size would be more recommended for this study.

**Conclusion:**

The reduction in the serum levels of IL-27 in vitiligo patients compared to normal subjects suggested the possible anti-inflammatory role of this cytokine in vitiligo. Thus, IL-27 may be considered as a new target for the manipulation of the immune system in vitiligo patients.

## Introduction

Vitiligo is a common acquired depigmentation disorder characterized by white patches which have been distributed in several parts of the body and greatly affects the quality of life and mental health of the patients.^1^, ^2^ The prevalence of vitiligo in different societies was published in a range of 0.5% to 1%.^3^ According to a recent meta-analysis, the prevalence of vitiligo among different communities was 0.2%, and from hospital-based studies, it was 1.8%.^4^ Vitiligo emerges with a notable pattern of macular depigmentation, with a variable appearance in shape or size. This disease is classified into two subgroups, generalized and localized, in both of them insufficiency of melanocyte function can be observed.^5^

The main causes of vitiligo are complex and maybe stem from genetic disorders, which can feature polygenic and multifactorial inheritance.^6^ The main cause is not yet clear; however, some evidence has shown that exchange in immunological programs contributed to this kind of action.^7^ Vitiligo is assumed to result from autoimmune reactions that gradually destroy melanocytes. Despite the detection of self-reacting auto-antibodies in vitiligo patients, increasing evidence supports impaired cellular immunity as the main cause of this disfiguring disease.^8^ Cytokines have a pivotal role as mediators of cellular and humoral immune reactions. The imbalance between pro- and anti-inflammatory cytokines favoring the dominance of a Th1/Th17 response rather than a Th2/Treg response has been proposed as a possible underlying mechanism of vitiligo. However, there is considerable evidence in support of the role of CD8 type 1 T cells in the destruction of melanocytes.^9^, ^10^ Increased expression of pro-inflammatory cytokines in vitiligo patients – including IL-1, IL-6, and TNF-α has been shown in several studies.^11^, ^12^, ^13^

IL-27, a rather new member of the IL-6/IL-12 family, is mainly released by activated antigen-presenting cells, including dendritic cells (DCs), monocytes, and macrophages.^14^, ^15^ It is composed of two subunits, EBV-induced 3 (EBI3), an IL-12 p40 homolog, and p28, an IL-6 p35 homolog. The IL-27 receptor (IL-27R) is a heterodimer composed of an IL-27R specific alpha chain (WSX-1) and a gp130 subunit.^16^ The IL-27R is expressed by several cell types including but not limited to T, B, and NK cells, DCs, macrophages, keratinocytes, CD8 type1 T-cells, and endothelial cells.^17^, ^18^, ^19^ IL-27R signaling results in the activation of JAK-STAT and p38 MAPK pathways.^20^, ^21^ IL-27 boosts the differentiation of Th1 and Tr1 cells through activation of STAT1/3 while inhibiting the differentiation of regulatory T-cells and Th2 cells.^21^, ^22^, ^23^, ^24^, ^25^, ^26^ Besides Th1 differentiation, IL-27 signaling through STAT-1 has also been demonstrated to activate NK cells, which have been considered to have a role in the pathogenesis of vitiligo.^27^

Although previous studies identified the inflammatory role of IL-27, the latest evidence suggests an immunomodulatory effect of this cytokine.^28^ IL-27 induces Tr1 and inhibits Th2 and Th17 responses, therefore limiting the severity of autoimmune diseases by the suppression of Th17 cells.^29^, ^30^, ^31^ It has also immune-regulatory functions due to the up-regulation of PD-L1, IDO, and IL-10.^32^ Thus, it has suggested that this cytokine may be considered as a possible therapeutic agent for some inflammatory or autoimmune diseases.

Regarding the complex pro-inflammatory and anti-inflammatory nature of IL-27, several studies have investigated the local or systemic concentrations of IL-27 in different diseases. Alteration in the concentration of IL-27 and its correlation with autoimmune parameters have also been reported in several Th1/Th17-mediated inflammatory disorders, such as multiple sclerosis, systemic lupus erythematosus, inflammatory bowel disease, and rheumatoid arthritis.^33^, ^34^, ^35^, ^36^

The concentrations of IL-27 in vitiligo patients have not yet been investigated. The present examination intended to evaluate the serum levels of IL-27 in vitiligo patients.

## Methods

### Study population

Seventy-nine vitiligo patients including 32 (41%) males and 47 (59%) females, and 45 age and sex-matched healthy controls with no sign of autoimmune diseases were enrolled in the study. The study population included 72 patients with generalized vitiligo and seven cases with localized vitiligo. Participants in this study assented to join according to the ethics committee of the medical school of Shiraz University of Medical Sciences (IR.sums.med.rec.1397.400).

### Sample collection

From all study populations, 5 mL of peripheral blood was collected. After centrifugation at 3000 rpm for 10 min, the sera were separated and kept at −70 °C until used.

### Measurement of serum IL-27

Serum concentrations of IL-27 were distinguished by enzyme-linked immunosorbent assay (ELISA) in vitiligo patients and healthy subjects. IL-27 concentrations were quantified using an ELISA kit (DY2526-05, R&D Systems – United States) manufacturers’ instructions. The sensitivity of tests was 12.8 pg/mL. Briefly, 100 μL of capture antibody was coated in each well of a 96-well microplate for overnight at room temperature (RT). After washing and blocking, 100 μL serum samples of patients and controls were added for 2 h, at RT. Plates were washed again and 100 μL of the detection antibody and then 100 μL of the working dilution of streptavidin horseradish peroxidase was added for 20 min. Then substrate solution (100 μL) was added for 20 min; finally, the reaction was stopped by adding stop solution to each well. The optical density of samples was read using a microplate reader at 450 nm. The levels of cytokine were extrapolated from the related standard curve.

### Statistical analysis

All data were analyzed by SPSS v. 16 software (SPSS Inc. – Chicago, IL, United States) and according to the normality test (Kolmogorov-Smirnov test); non-parametric (Mann–Whitney *U* and Kruskal–Wallis) tests were used; *p*-values < 0.05 were considered as significant. Graphs were constructed using Graph Pad Prism (v. 6 – La Jolla, CA, United States). All data are presented as mean ± standard error of the mean (SEM), unless otherwise specified.

## Results

### Demographic analysis

An overview of the demographic and clinical features of vitiligo patients and healthy subjects participated in the study is provided in Table 1.

**Table 1:** Demographic analysis of population included in this study

Study population	Patients	Controls
*Number*	79	45
*Age*	36.37 ± 14.7	35.06 ± 11.5

*Sex*		
Male	32 (41%)	18 (40%)
Female	47 (59%)	27 (60%)

### Measurement of serum IL-27

**Figure 1:**
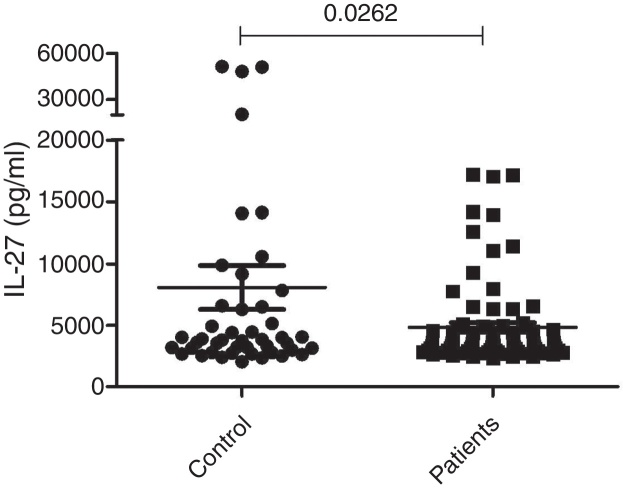
Mean serum concentration of IL-27 (pg/mL) in vitiligo patients and control group.

The serum concentrations of IL-27 in 79 patients with generalized (72 cases) or localized (seven cases) vitiligo were observed using the ELISA technique. Forty-five age and sex-matched healthy subjects were used as the control group. As presented in Figure 1, the results showed that there was a significant difference in serum concentrations of IL-27 between vitiligo patients (5267.8 ± 399 pg/mL) and healthy subjects (7097.7 ± 1502 pg/mL) (*p* = 0.0262).

### Correlations of IL-27 with demographic information and clinical manifestations

The correlations between serum concentrations of IL-27 with gender, the severity of the disease, two types of (localized/generalized) vitiligo, segmental/nonsegmental forms of the disease, and response to treatment in patients with vitiligo were examined. There was no significant correlation between IL-27 levels and these parameters in the present study (Table 2).

**Table 2:** Comparison of serum concentrations of IL-27 based on demographic and clinical manifestations in vitiligo patients

		*n*	Mean (pg/mL)	*p*-Value
Gender	Male	32	4623.8 ± 544.2	0.681
	Female	47	4961.5 ± 564.5	
	Total	79		

Severity (spread of lesions)	Body area <20%	49	4793.9 ± 486.6	0.071
	Body area 20%–50%	24	4132.7 ± 435.1	
	Body area >50%	6	7844.7 ± 2928.6	
	Total	79		

Type: localized or generalized	Generalized	72	4925.9 ± 434.3	0.421
	Localized	7	3784.3 ± 541.5	
	Total	79		

Type: segmental or non-segmental	Segmental	8	3668.8 ± 483.0	0.335
	Non-segmental	71	4955.0 ± 439.5	
	Total	79		

Response to treatment	Yes	57	4492.8 ± 380.9	0.154
	No	21	5797.1 ± 1083.9	
	Total	78		

## Discussion

The main reason for vitiligo is ill defined. Human and experimental studies have recently provided considerable evidence regarding the pattern of autoimmunity in the pathogenesis of vitiligo. Both humoral and cellular immunity have shown to be involved in the etiology of the disease.^37^, ^38^, ^39^ The accumulated evidence suggests a primary role for cell-mediated mechanisms, including Th1/Th17 and Tc1 cells in the pathogenesis of vitiligo.^40^ Various inflammatory cytokines (IL-1, IL-6, TNF-α, IL-6, and IL-17) have a key role in skin depigmentation, while the level of TGF-β indicated reversible state.^6^, ^41^

IL-27 has been suggested to function as a pro-inflammatory as well as an anti-inflammatory cytokine. Altered concentrations of IL-27 have been shown in several autoimmune and skin disorders. Despite early studies identified pro-inflammatory role of IL-27, latest evidence suggests that it suppresses a range of immune cell proliferation and cytokine production.^28^ These cytokine applications are related to its pro- or anti-inflammatory activity. The dual role of IL-27 is related to the different tissues involved, the underlying mechanism, or the kind and stage of autoimmune diseases.^29^ Previous studies revealed that the blood concentration of IL-27 increased in pemphigus and psoriatic patients. They showed that IL-27 levels strongly correlated with the IgG auto-antibody titers in pemphigus, and also disease onset and severity of psoriasis.^42^, ^43^ The expression of IL-27 in lesional eczematous skin has been reported too.^43^ This evidence supports a pro-inflammatory function of IL-27. In spite of skin disorders in which IL-27 plasma concentrations have increased, the serum level of this cytokine is reduced in some auto-immune diseases such as Vogt-Koyanagi-Harada syndrome (VKH), Behçet's disease (BD), and SLE. Wang and his colleagues described that the expression of IL-27 p28 mRNA by peripheral blood mononuclear cells (PBMCs) and serum concentration of IL-27 in the sera and supernatants of cultured PBMCs were noticeably reduced in patients with active BD and VKH. In addition, Gaber et al. have observed that the IL-27 level in SLE patients is markedly lower than in healthy controls.^44^, ^45^, ^46^

Based on those explanations and the results of several studies about the altered expression of IL-27 in autoimmunity and skin disorders, it was hypothesized that the concentration of IL-27 in vitiligo patients might be altered. Consistent with previous studies in SLE and BD patients, the present study showed that the concentrations of IL-27 serum levels were reduced in vitiligo patients, which can be justified through some probable mechanisms. First, IL-27 acts against Th17 development in a direct manner through modulating DCs and induces IL-10 production by naïve CD4+ T-cells.^45^ Th17 contributes to the pathogenesis of vitiligo by IL-17 production. IL-27 inhibits the differentiation and generation of Th17 cells via IL-6 and transforming growth factor-β (TGF-β) suppression, which is dependent on the intracellular signaling molecule STAT1.^44^

## Conclusion

It can be concluded that IL-27 has an immunomodulatory role in vitiligo. According to the observations of the present study, there was no association between IL-27 serum level and demographic information, severity of the disease, types of vitiligo, and response to treatment in patients with vitiligo. Further studies with more patients of all types of vitiligo are needed to reveal the possible alterations in the concentration of IL-27 in those patients and their correlation with disease characteristics, including severity and the extension of the affected skin area. In conclusion, IL-27 has two roles; it should be considered as a new target for the manipulation of the immune system in various immune-mediated disorders.

## Financial support

This study was supported by 10.13039/501100004320Shiraz University of Medical Sciences (grant No. 17684).

## Authors’ contributions

Saeed Malek Hosseini: Drafting and editing of the manuscript.

Naser Gholijani: Collection, analysis, and interpretation of data; participation in the study design.

Nooshafarin Chenari: Collection, analysis, and interpretation of data; participation in the study design.

Nooshafarin Chenari: Drafting and editing of the manuscript.

## Conflicts of interest

None declared.

## References

[1] Nordlund J.J. (2011). Vitiligo: a review of some facts lesser known about depigmentation. Indian J Dermatol.

[2] Parsad D., Dogra S., Kanwar A.J. (2003). Quality of life in patients with vitiligo. Health Qual Life Outcomes.

[3] Lee H., Lee M.H., Lee D.Y., Kang H.Y., Kim K.H., Choi G.S. (2015). Prevalence of vitiligo and associated comorbidities in Korea. Yonsei Med J.

[4] Zhang Y., Cai Y., Shi M., Jiang S., Cui S., Wu Y. (2016). The prevalence of vitiligo: a meta-analysis. PLOS ONE.

[5] Boniface K., Seneschal J., Picardo M., Taïeb A. (2018). Vitiligo: focus on clinical aspects, immunopathogenesis, and therapy. Clin Rev Allergy Immunol.

[6] Sandoval-Cruz M., García-Carrasco M., Sánchez-Porras R., Mendoza-Pinto C., Jiménez-Hernández M., Munguía-Realpozo P. (2011). Immunopathogenesis of vitiligo. Autoimmun Rev.

[7] Laddha N.C., Dwivedi M., Mansuri M.S., Gani A.R., Ansarullah M., Ramachandran A.V. (2013). Vitiligo: interplay between oxidative stress and immune system. Exp Dermatol.

[8] van den Boorn J.G., Konijnenberg D., Dellemijn T.A., van der Veen J.P., Bos J.D., Melief C.J. (2009). Autoimmune destruction of skin melanocytes by perilesional T cells from vitiligo patients. J Invest Dermatol.

[9] Wu J., Zhou M., Wan Y., Xu A. (2013). CD8+ T cells from vitiligo perilesional margins induce autologous melanocyte apoptosis. Mol Med Rep.

[10] Ogg G.S., Rod Dunbar P., Romero P., Chen J.L., Cerundolo V. (1998). High frequency of skin-homing melanocyte-specific cytotoxic T lymphocytes in autoimmune vitiligo. J Exp Med.

[11] Singh S., Singh U., Pandey S.S. (2012). Serum concentration of IL-6, IL-2, TNF-α, and IFNγ in vitiligo patients. Indian J Dermatol.

[12] Sushama S., Dixit N., Gautam R.K., Arora P., Khurana A., Anubhuti A. (2019). Cytokine profile (IL-2, IL-6, IL-17, IL-22, and TNF-alpha) in vitiligo – new insight into pathogenesis of disease. J Cosmet Dermatol.

[13] Singh M., Mansuri M.S., Mansi A. (2016). Parasrampuria and Rasheedunnisa Begum. Interleukin 1-α: a modulator of melanocyte homeostasis in vitiligo.

[14] Pflanz S., Timans J.C., Cheung J., Rosales R., Kanzler H., Gilbert J. (2002). IL-27, a heterodimeric cytokine composed of EBI3 and p28 protein, induces proliferation of naive CD4+ T cells. Immunity.

[15] Morrow K.N., Coopersmith C.M., Ford M.L. (2019). IL-17, IL-27, and IL-33: A novel axis linked to immunological dysfunction during sepsis. Front Immunol.

[16] Kastelein R.A., Hunter C.A., Cua D.J. (2007). Discovery and biology of IL-23 and IL-27: related but functionally distinct regulators of inflammation. Annu Rev Immunol.

[17] Ruckerl D., Hessmann M., Yoshimoto T., Ehlers S., Hölscher C. (2006). Alternatively activated macrophages express the IL-27 receptor alpha chain WSX-1. Immunobiology.

[18] Hunter C.A., Kastelein R. (2012). Interleukin-27: balancing protective and pathological immunity. Immunity.

[19] Meka R.R., Venkatesha S.H., Dudics S., Acharya B., Moudgil K.D. (2015). IL-27-induced modulation of autoimmunity and its therapeutic potential. Autoimmun Rev.

[20] Charlot-Rabiega P., Bardel E., Dietrich C., Kastelein R., Devergne O. (2011). Signaling events involved in interleukin 27 (IL-27)-induced proliferation of human naive CD4+ T cells and B cells. J Biol Chem.

[21] Guzzo C., Che Mat N.F., Gee K. (2010). Interleukin-27 induces a STAT1/3- and NF-kappaB-dependent proinflammatory cytokine profile in human monocytes. J Biol Chem.

[22] Wang H., Meng R., Li Z., Yang B., Liu Y., Huang F. (2011). IL-27 induces the differentiation of Tr1-like cells from human naive CD4+ T cells via the phosphorylation of STAT1 and STAT3. Immunol Lett.

[23] Artis D., Villarino A., Silverman M., He W., Thornton E.M., Mu S. (2004). The IL-27 receptor (WSX-1) is an inhibitor of innate and adaptive elements of type 2 immunity. J Immunol.

[24] Diveu C., McGeachy M.J., Boniface K., Stumhofer J.S., Sathe M., Joyce-Shaikh B. (2009). IL-27 blocks RORc expression to inhibit lineage commitment of Th17 cells. J Immunol.

[25] El-behi M., Ciric B., Yu S., Zhang G.X., Fitzgerald D.C., Rostami A. (2009). Differential effect of IL-27 on developing versus committed Th17 cells. J Immunol.

[26] Schneider R., Yaneva T., Beauseigle D., El-Khoury L., Arbour N. (2011). IL-27 increases the proliferation and effector functions of human naive CD8+ T lymphocytes and promotes their development into Tc1 cells. Eur J Immunol.

[27] Ziblat A., Domaica C.I., Spallanzani R.G., Iraolagoitia X.L., Rossi L.E., Avila D.E. (2015). IL-27 stimulates human NK-cell effector functions and primes NK cells for IL-18 responsiveness. Eur J Immunol.

[28] Villarino A.V., Huang E., Hunter C.A. (2004). Understanding the pro-and anti-inflammatory properties of IL-27. J Immunol.

[29] Fabbi M., Carbotti G., Ferrini S. (2017). Dual roles of IL-27 in cancer biology and immunotherapy. Mediators Inflamm.

[30] Pot C., Apetoh L., Awasthi A., Kuchroo V.K. (2011). Kuchroo. Induction of regulatory Tr1 cells and inhibition of TH17 cells by IL-27. Semin Immunol.

[31] Stumhofer J.S., Hunter C.A. (2008). Advances in understanding the anti-inflammatory properties of IL-27. Immunol Lett.

[32] Awasthi A., Carrier Y., Peron J.P., Bettelli E., Kamanaka M., Flavell R.A. (2007). A dominant function for interleukin 27 in generating interleukin 10-producing anti-inflammatory T cells. Nat Immunol.

[33] Sénécal V., Deblois G., Beauseigle D., Schneider R., Brandenburg J., Newcombe J. (2016). Production of IL-27 in multiple sclerosis lesions by astrocytes and myeloid cells: modulation of local immune responses. Glia.

[34] Gaber W., Sayed S., Rady H.M., Mohey A.M. (2012). Interleukin-27 and its relation to disease parameters in SLE patients. Egypt Rheumatol.

[35] Lai X., Wang H., Cao J., Li Y., Dai Y., Xiang Y. (2016). Circulating IL-27 is elevated in rheumatoid arthritis patients. Molecules.

[36] Schmidt C., Giese T., Ludwig B., Mueller-Molaian I., Marth T., Zeuzem S. (2005). Expression of interleukin-12-related cytokine transcripts in inflammatory bowel disease: elevated interleukin-23p19 and interleukin-27p28 in Crohn's disease but not in ulcerative colitis. Inflamm Bowel Dis.

[37] Ongenae K., Van Geel N., Naeyaert J.M. (2003). Evidence for an autoimmune pathogenesis of vitiligo. Pigment Cell Res.

[38] Hann S.K., Shin H.K., Park S.H., Reynolds S.R., Bystryn J.C. (1996). Detection of antibodies to melanocytes in vitiligo by western immunoblotting. Yonsei Med J.

[39] Alikhan A., Felsten L.M., Daly M., Petronic-Rosic V. (2011). Vitiligo: a comprehensive overview. Part I. Introduction, epidemiology, quality of life, diagnosis, differential diagnosis, associations, histopathology, etiology, and work-up. J Am Acad Dermatol.

[40] Kalaiselvi R., Rajappa M., Chandrasekhar L., Thappa D.M., Munisamy P. (2019). Immunophenotype of circulatory T-helper cells in patients with non-segmental vitiligo. Postepy Dermatol Alergol.

[41] Gholijani N., Yazdani M.R., Dastgheib L. (2020). Predominant role of innate pro-inflammatory cytokines in vitiligo disease. Arch Dermatol Res.

[42] Hennerici T., Pollmann R., Schmidt T., Seipelt M., Tackenberg B., Möbs C. (2016). Increased frequency of t follicular helper cells and elevated interleukin-27 plasma levels in patients with pemphigus. PLOS ONE.

[43] Shibata S., Tada Y., Kanda N., Nashiro K., Kamata M., Karakawa M. (2010). Possible roles of IL-27 in the pathogenesis of psoriasis. J Invest Dermatol.

[44] Gaber W., Sayed S., Rady H.M., Mohey A.M. (2012). Interleukin-27 and its relation to disease parameters in SLE patients.

[45] Wang C., Tian Y., Lei B., Xiao X., Ye Z., Li F. (2012). Decreased IL-27 expression in association with an increased Th17 response in Vogt-Koyanagi-Harada disease. Invest Ophthalmol Vis Sci.

[46] Wang C., Tian Y., Ye Z., Kijlstra A., Zhou Y., Yang P. (2014). Decreased interleukin 27 expression is associated with active uveitis in Behçet's disease. Arthritis Res Ther.

